# Effect of micro-strain stress on in vitro proliferation and functional expression of human osteoarthritic chondrocytes

**DOI:** 10.1186/s13018-022-02987-9

**Published:** 2022-02-15

**Authors:** Bin Zhao, Jianxiong Ma, Jinquan He, Xinlong Ma

**Affiliations:** Institute of Orthopedics, Tianjin Hospital, Tianjin, China

**Keywords:** Osteoarthritis, Chondrocyte, Micro-strain stress, Proliferation, Functional expression

## Abstract

**Background:**

This study aimed to analyze the in vitro effect of micro-strain stress on the proliferation and functional marker expression in chondrocytes isolated from human osteoarthritis cartilage samples.

**Methods:**

Chondrocytes isolated from human osteoarthritis cartilage samples were subjected to loading with different types of micro-strain stress. The proliferation activity was assessed by flow cytometry, and the functional expression of chondrocyte markers was detected by qRT-PCR and western blot.

**Results:**

Flow cytometry results showed stimulation of proliferation of human osteoarthritic chondrocytes when an adequate micro-strain stress was applied. qRT-PCR and western blot results showed that micro-strain stress promotes human osteoarthritic chondrocyte functional marker expression. These features coincide with the upregulation of multiple proteins and genes affecting cell proliferation and functional chondrocyte marker expression, including cyclin D1, collagen II, and Rock.

**Conclusion:**

Adequate micro-strain stress could activate the Rho/Rock signaling pathway in osteoarthritic chondrocytes, thus transmitting mechanical signals to the cytoskeleton. This process leads to cytoskeleton reorganization, and transmission of the mechanical signals to the downstream effectors to promote proliferation and functional marker expression of osteoarthritic chondrocytes.

## Introduction

Osteoarthritis is the most common type of joint disease, and its pathology is characterized by chondrocyte apoptosis and cartilage matrix destruction [1–3]. Cartilage tissue, which covers the joint surface, is a specialized connective tissue without blood vessels. It is stimulated continuously by endogenous and exogenous mechanical stimuli. Mechanical stimulation in a specific range plays a critical role in the maintenance of the structural integrity of articular cartilage [4]. Some in vitro studies have shown that loading cultured chondrocytes with different types of mechanical stimulation could produce varying degrees of cell biological changes [5–7].

The cellular mechanism of how osteoarthritic chondrocytes sense mechanical stress stimulation signals and transfer them into the cells to regulate bone remodeling is not thoroughly understood. The cytoskeleton is a critical component involved in the maintenance of cell morphology and various cellular functions [8, 9]. After mechanical stimulation, the cytoskeletal structures undergo dynamic rearrangement, polymerization, and depolymerisation [10]. The cytoskeleton plays a vital role in the maintenance of the intracellular structures and could rapidly transmit stress from the cytoskeleton to effectors in cells, thus causing various biological effects [11, 12]. In studies on vascular endothelial cells, osteoblasts, and bone marrow mesenchymal stem cells, it has been shown that cytoskeletal-mediated mechanical signaling plays a crucial role in the reactivity of various cell lines to mechanical stimulation [13]. At present, the biological effects of stress stimulation on osteoarthritic chondrocytes and the underlying role of the cytoskeleton are still unclear, and further experimental studies are needed urgently.

In this study, the varying extent of micro-strain stress was applied to cultured human osteoarthritic chondrocytes and their effects on cell proliferation and functional expression of markers were evaluated. The regulatory effect of differing micro-strain stress on the cytoskeleton of osteoarthritic chondrocytes was studied by confocal fluorescence microscopy to explore the underlying role of the cytoskeleton in mediating the mechanical stress signals to regulate the physiological functions of osteoarthritic chondrocytes. The role of the Rho/Rock signaling pathway in the above process was studied through the use of the Rho/Rock signaling pathway inhibitor Y-27632.

## Materials and methods

### Study objects

After obtaining the informed consent from patients with osteoarthritis of the knee, a small sample of cartilage tissue was removed during surgery. Tissues were collected aseptically, and sections prepared for Hematoxylin and Eosin (H&E) staining. Chondrocytes were isolated from the remaining tissues and cultured within two hours.

The Ethical Committee of Tianjin Hospital approved this study. Following the Declaration of Helsinki guidelines, consents were obtained from either the patient or family member before enrollment in the study.

### Isolation, culture, and identification of chondrocytes

The tissue was soaked in D-Hank's solution containing antibiotics for 5 min and rinsed twice with D-Hank's solution to remove the blood and fat from the cartilage tissue. The tissue was cut into 1 × 1 × 1 mm^3^ sized tissue blocks and digested with 0.02% EDTA and 0.25% trypsin at 37℃ for 30 min. The trypsin–EDTA solution was removed, after which 0.2% collagen II enzyme was added, and the tissue digested overnight at 4℃. The tissue was digested almost completely after incubation at 37℃ for 2 h, and the addition of 1 ml FBS stopped the digestion. The resulting sample was filtered using a 200-mesh filter, and the filtrate transferred to a tube and centrifuged at 1500 r/min for 7 min, and the supernatant discarded. The cells were suspended in an H-DMEM medium containing 10% FBS, and cells counted. A 25 cm^2^ cell culture flask was inoculated with 5 × 10^4^/ml of cells. After 24 h, half the volume was replaced with fresh media changes every two days. When the cells reached 80–90% confluence, the cells were trypsinized and passaged.

The cells derived were identified by toluidine blue staining, Safranin O staining, and collagen II immunofluorescence staining. The experimental methods were carried out following the kit instructions. Cells at passage three were used for subsequent cell mechanical loading tests.

### Cell mechanical loading

We performed the mechanical loading of cells following the methods described earlier by our group [14–16]. The conditions used were: mechanical loading frequency, 0.25 HZ; time, 2 h/day; duration, three days. The study was divided into five groups: group A, 0% micro-strain; group B, 5% micro-strain; group C, 10% micro-strain; group D, 15% micro-strain; group E, 10% micro-strain plus Rock inhibitor Y-27632.

### Immunofluorescence detection

Cells of each group were washed with phosphate-buffered saline (PBS) and fixed in 4% paraformaldehyde. Following fixation, the cells were washed with PBS and permeabilized with Triton X-100. After washing with PBS, the cells were blocked with 1% goat serum and incubated at room temperature with FITC labeled phalloidin. After PBS wash, the cells were stained with DAPI, and the immunofluorescent cytoskeletal proteins were observed under confocal microscopy.

### Cell cycle analysis

The cell cycle analysis was performed as described previously [15]. After mechanical loading, cells from each group were cultured in the incubator for another 24 h, allowing adequate recovery time. The cells were digested enzymatically, centrifuged at 2,000 r/min for 5 min, and the supernatant discarded. The cells were rinsed twice with sterile D-Hank’s solution and fixed in 75% cold ethanol. The cells were centrifuged at 2,000 r/min for 5 min, and 500 μl RNAase was added to the cell pellet and incubated at 37℃ for 30 min. The cells were centrifuged at 2,000 r/min for 5 min, and 500 μl propidium iodide (PI) was added and incubated at room temperature in the dark for 30 min. The cell cycle analysis was carried out by flow cytometry. The data were calculated by Flow Plus software. The proliferative index, the proportion (%) of cells in the S phase were calculated based on the G0/G1 phase and G2/M phase, using the formula: S(%) = 1 − [G0/G1(%) + G2/M(%)], PI(%) = S(%) + G2/M(%).

### Quantitative real-time (qRT) PCR

We extracted total RNA from each group of cells using Trizol following the manufacturer’s instructions. The total RNA was reverse transcribed to cDNA using Prime Script RT reagent kit. The primers used for the genomic analysis of the chondrocytes were as follows: β-actin, F 5′-CCTCGCCTTTGCCGATCC-3′ and R 5′-GGATCTTCATGAGGTAGTCAGTC-3′; cyclin D1, F 5′-AAAGGAAGCAAGAACCCAT-3′ and R 5′-GTCCGAGATTATCATTACCC-3′; collagen II, F 5′-TTCAGCTATGGAGATGACAATC-3′ and R 5′-AGAGTCCTAGAGTGACTGAG-3′. The reaction conditions were as outlined in the SYBR Premix Ex Taq. The amplification and melting curves were analyzed after the reaction was completed. Gene expression values were analyzed for target gene expression by the 2^−△△Ct^ method.

### Western blot analysis

We extracted total proteins from each group of cells using RIPA lysis method. The proteins were quantified by BCA protein quantitation kit. An equal amount of protein from each group was separated on SDS-PAGE and transferred to nitrocellulose (NC) membranes. After blocking with 5% milk, the NC membranes were incubated with primary antibodies against β-actin, cyclin D1, collagen II, and Rock, followed by appropriate HRP-conjugated secondary antibodies. The membranes were treated with ECL, and bands were visualized.

### Statistical analysis

SPSS 18.0 software was used for analysis. Data were expressed as mean ± standard deviation (*x* ± *s*), one-way ANOVA was used for multi-group comparison, and the LSD-t method was used for multi-group comparison. *P* < 0.05 was considered statistically significant.

## Results

### Characterization and identification of cartilage and chondrocytes

**Fig. 1:**
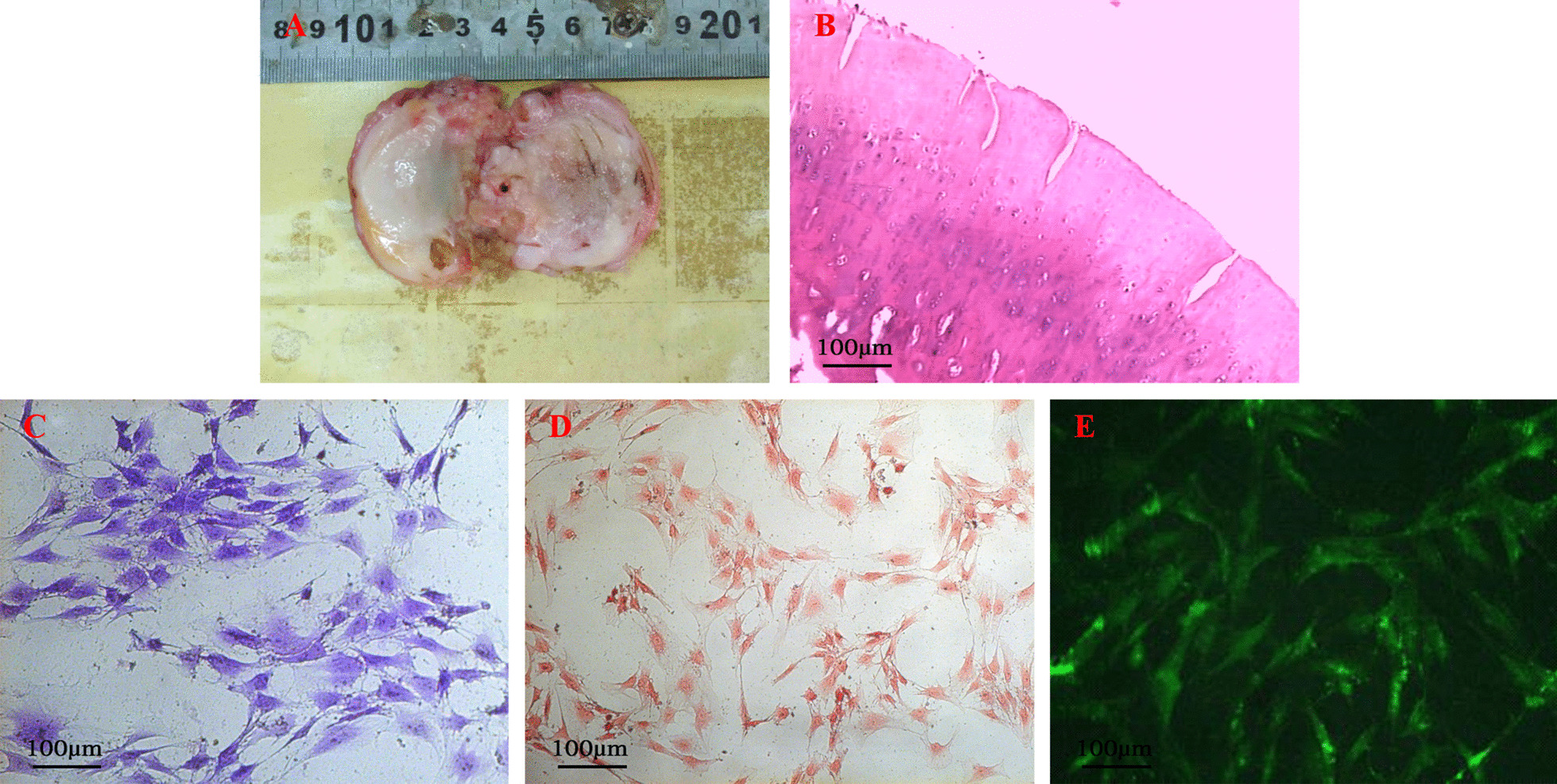
Characterization and identification of cartilage and chondrocytes. **A** Gross view of knee osteoarthritis cartilage. **B** The H&E staining of knee osteoarthritis cartilage. **C** Toluidine blue staining of osteoarthritic chondrocytes. **D** Safranin O staining of osteoarthritic chondrocytes. **E** Collagen II immunofluorescence staining of osteoarthritic chondrocytes

Gross observation: the articular cartilage lost its original luster, and its color darkened markedly. Articular cartilage surface defects of the lateral femoral condyle were noted, and subchondral bone was exposed, as shown in Fig. 1A. H&E staining showed the formation of longitudinal cracks in the cartilage, showing a fibrillation-like change and disordered cell arrangement. In the deep layer of the cartilage, cell aggregation, as shown in Fig. 1B was observed. The results, when combined with the clinicopathological data of the patient, showed moderate and severe degeneration of the articular cartilage.

Toluidine blue staining results showed blue cytoplasm, confirming that cells secrete glycosaminoglycan (Fig. 1C). Safranin O staining results showed red cytoplasm, thus identifying than cells secrete proteoglycan (Fig. 1D). Collagen II immunofluorescence staining results showed green fluorescent cytoplasm suggesting secretion of collagen II (Fig. 1E). The above results confirmed that the cultured cells were chondrocytes.

### Micro-strain stress causes reorganization of osteoarthritic chondrocyte cytoskeleton

**Fig. 2:**
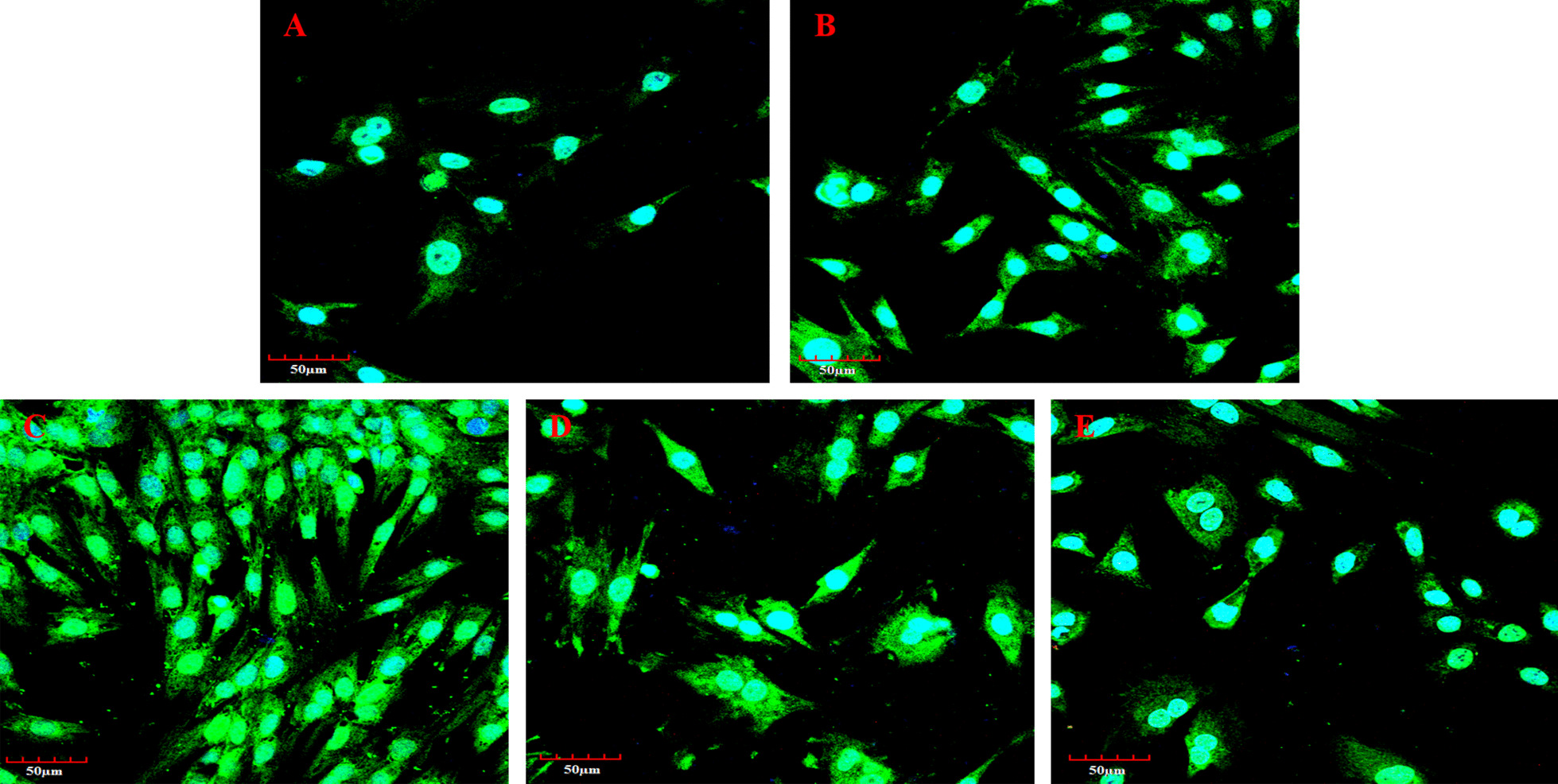
Effects of micro-strain stress on the expression of osteoarthritic chondrocyte cytoskeleton protein. **A**–**E** show the phalloidin immunofluorescence staining of each group

The expression and distribution of cytoskeletal proteins in chondrocytes under micro-strain stress were evaluated by immunofluorescence, and the images captured by confocal microscopy: cells in group A showed uniform fluorescence staining, scattered and distributed in the cytoplasm without any directionality (Fig. 2A). The cells in group B showed a fine fiber staining pattern, distributed in a specific direction (Fig. 2B). The cells in group C showed intense fluorescence staining, and the actin fibers were relatively thick, evenly distributed and arranged in bundles in parallel, and distributed in the direction of stress (Fig. 2C). The cells in group D were composed mainly of fine fibers, which were distributed in a specific direction (Fig. 2D). The above results suggested that the application of adequate micro-strain stress could lead to the reorganization of the cytoskeleton.

### Micro-strain stress promotes the proliferation of osteoarthritic chondrocytes

**Fig. 3:**
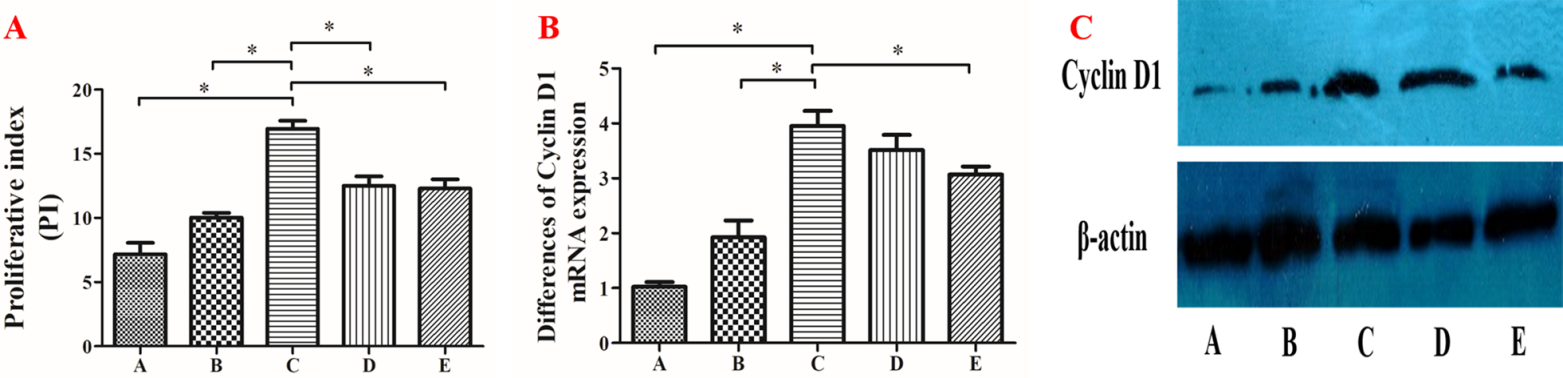
Effects of micro-strain stress on the proliferation of osteoarthritic chondrocytes. **A** Proliferative index of osteoarthritic chondrocytes after different micro-strain stress for 3-days (*P < 0.05). **B** Cyclin D1 mRNA expression of osteoarthritic chondrocytes after different micro-strain stress for 3-days (*P < 0.05). **C** Cyclin D1 protein expression of osteoarthritic chondrocytes after different micro-strain stress for 3-days

Cell cycle analysis by flow cytometry was used to examine the micro-strain stress effect on the proliferation of osteoarthritic chondrocytes. PI staining shows the ability of cells to proliferate. With increased micro-strain stress, PI staining increased gradually, reaching peak levels in group C, and decreased gradually (Fig. 3A). Based on the qRT-PCR results, changes in Cyclin D1 gene expression were similar to changes in PI staining. With increasing micro-strain stress, the Cyclin D1 gene expression increased gradually, and reached a peak in group C, then progressively decreased (Fig. 3B). Western blot results showed that with an increase in micro-strain stress, Cyclin D1 protein expression increased gradually, and peaked in group C, then decreased gradually (Fig. 3C). These results suggested that adequate micro-strain stress could promote the proliferation of osteoarthritic chondrocytes.

### Micro-strain stress promotes the functional expression of osteoarthritic chondrocyte markers

**Fig. 4:**
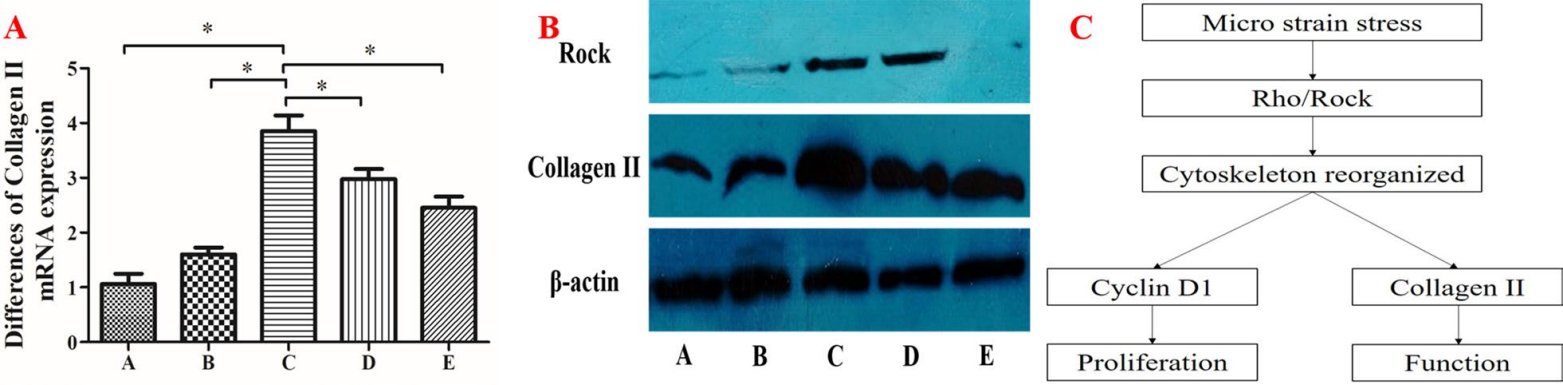
Effects of micro-strain stress on functional marker expression in osteoarthritic chondrocytes. **A** Collagen II mRNA expression in osteoarthritic chondrocytes after different micro-strain stress for 3-days (*P < 0.05). **B** Collagen II and Rock protein expression in osteoarthritic chondrocytes after different micro-strain stress for 3-days. **C** Schematic diagram of possible signaling pathways

The effects of micro-strain stress on functional expression of osteoarthritic chondrocyte markers were examined by detecting the collagen II gene and protein expression in each group. qRT-PCR results showed that with increasing micro-strain stress, collagen II gene expression increased gradually, and reached a peak in group C, then steadily decreased (Fig. 4A). Western blot results showed a gradual increase in collagen II protein expression with increased micro-strain stress, which reached a peak in group C, then decreased gradually (Fig. 4B). These results showed the functional expression of osteoarthritic chondrocyte markers after the application of adequate micro-strain stress.

### Expected mechanism

Phalloidin immunofluorescence results showed that compared to group C, the crude fibers in group E cells disappeared and were replaced by tiny fibers, which were distributed diffusely in the cytoplasm with no directionality (Fig. 2E). The cell cycle results showed a lower proliferative index value in group E compared to group C (*P* < 0.05, Fig. 3A). The cyclin D1 and collagen II gene and protein expression in group E were lower compared to group C as shown by qRT-PCR and western blot results (*P* < 0.05, Fig. 3B, C) and (*P* < 0.05, Fig. 4A, B), respectively. Western blot results showed a high expression of Rock in groups C and D, moderate expression in group B, and low expression in groups A and C (Fig. 4B). These results revealed the involvement of the Rho/Rock signaling pathway in the above biological changes (Fig. 4C).

## Discussion

The present study used a novel micro-strain stress loading system designed by our research group [14–16]. The osteoarthritic chondrocyte proliferation and functional marker expression were detected after subjecting cells to varying micro-strain stress during culture. The results showed that the application of adequate micro-strain stress could promote osteoarthritic chondrocyte proliferation and functional marker expression. The cyclin D1 and collagen II mRNA and protein expression increased significantly under micro-strain stress and peaked in the 10% micro-strain group. With increasing micro-strain stress, the cytoskeleton exhibited rearrangement. However, the above-described changes were suppressed when the Rock inhibitor Y-27632 was used before the application of micro-strain stress.

Articular cartilage is in a complex physiological and mechanical environment in the body [17, 18]. The mechanical stimuli are important factors in maintaining the normal structure and function of articular cartilage [19, 20]. Xu et al. [21] applied intermittent cyclic mechanical tension (0.5 Hz, 10% deformation, 4 h/d, 6d/week) to rat endplate chondrocytes, and showed that tension stimulation promotes the proliferation of endplate chondrocytes. Thomopoulos et al. [22] applied cyclic tensile loading tension (1 Hz, 10% deformation, 7d) to bone marrow stromal cells in a 3D in vitro model, showing that the cyclic tensile strain could promote spindle cell formation, increase collagen I and glycosaminoglycan synthesis. Therefore, we believe that an appropriate micro-strain stress could promote the proliferation and matrix anabolism of chondrocytes, and also maintain the normal structure and function of chondrocytes. However, micro-strain stress beyond the chondrocytes bearing range might inhibit cell proliferation, damage structural function and integrity of cells, and further damage the articular cartilage. Such changes weaken the ability of damaged cartilage tissue to withstand mechanical stimulation and may further aggravate the effects of mechanical stimulation leading to a vicious cycle of complete loss of cartilage tissue structure and function.

The experimental results showed that proliferative index values, mRNA, and cyclin D1 and collagen II protein expression increased gradually with increasing micro-strain stress, and peaked at 10% micro-strain stress and then decreased gradually. However, after pretreatment of cells with the Rock inhibitor Y-27632, the proliferative index values, mRNA, and cyclin D1 and collagen II protein expression decreased. The above results indicate that micro-strain stress affects the proliferation and functional marker expression of osteoarthritic chondrocytes, and 10% micro-strain stress might offer the best mechanical stimulation. Rock inhibitor Y-27632 inhibits this process.

After experiencing mechanical stimulation, the cells convert mechanical signals to chemical signals through specific signal transduction mechanisms resulting in changes in the biological function [23, 24]. In this series of signal transduction processes, the cytoskeleton plays a crucial role as the hub across the cell [25]. Cytoskeleton, a critical component of cells, is composed of a large number of actin filaments and is the internal framework of cells [26]. It consists of microtubules, microfilaments, and intermediate filaments, which are interlinked with protein-lipid molecules of the cytoplasmic side of the cell membrane to form the structural basis for cell movement, cell morphology, and transmembrane information transmission [27]. It was shown earlier that cytoskeletal rearrangements occur during periodic mechanical stress and space microgravity [28]. Phalloidin specifically binds to actin fibers in the cytoskeleton [29]. The results of this experiment confirmed that under appropriate micro-strain stress, the osteoarthritic chondrocyte cytoskeletal microfilaments changed in structure and arrangement. However, too much mechanical stimulation can suppress the above changes.

Studies have shown that Rho GTPases can regulate the structure and function of the cytoskeleton in several ways under biomechanical stimulation, thus playing a critical role in biomechanical signal transduction [30, 31]. Rock is the downstream signaling molecule of the Rho GTP family and plays a crucial role in the Rho signaling pathway [32]. Y-27632 has been widely used as an inhibitor of the Rho/ROCK signaling pathway [33–35]. Therefore, Rock specific inhibitor Y-27632 was selected in this study. Whether the Rho/Rock signaling pathway is involved in the regulation of osteoarthritic chondrocyte proliferation and functional expression of markers induced by micro-strain stress is still unclear. Western blot results showed that with increasing micro-strain stress, the Rock level increased gradually, reached a peak at 10% micro-strain, and decreased later. These results showed activation of the Rho/Rock signaling pathway in osteoarthritic chondrocytes by micro-strain stress, leading to cytoskeletal reorganization, and promoting the proliferation and functional expression of osteoarthritic chondrocyte markers.

In summary, adequate micro-strain stress can activate the Rho/Rock signaling pathway in osteoarthritic chondrocytes, which leads to the transmission of mechanical signals to the cytoskeleton. The above processes cause the cytoskeletal reorganization and transmit the mechanical signals, leading to the promotion of proliferation and functional expression of osteoarthritic chondrocyte markers.

## Data Availability

All data generated during this study are included in this manuscript.

## Abbreviations

H&E staining: Hematoxylin and Eosin staining
PBS: Phosphate-buffered saline
PI: Propidium iodide
NC: Nitrocellulose
